# Bcl-2-dependent autophagy disruption during aging impairs amino acid utilization that is restored by hochuekkito

**DOI:** 10.1038/s41514-021-00065-8

**Published:** 2021-07-01

**Authors:** Miwa Nahata, Sachiko Mogami, Hitomi Sekine, Seiichi Iizuka, Naoto Okubo, Naoki Fujitsuka, Hiroshi Takeda

**Affiliations:** 1 Tsumura Kampo Research Laboratories, Tsumura & Co., Ibaraki, Japan; 2 Pathophysiology and Therapeutics, Faculty of Pharmaceutical Sciences, Hokkaido University, Sapporo, Hokkaido Japan; 3 Hokkaido University Hospital Gastroenterological Medicine, Sapporo, Hokkaido Japan

**Keywords:** Autophagy, Nutrition, Mouse

## Abstract

Chronic undernutrition contributes to the increase in frailty observed among elderly adults, which is a pressing issue in the sector of health care for older people worldwide. Autophagy, an intracellular recycling system, is closely associated with age-related pathologies. Therefore, decreased autophagy in aging could be involved in the disruption of energy homeostasis that occurs during undernutrition; however, the physiological mechanisms underlying this process remain unknown. Here, we showed that 70% daily food restriction (FR) induced fatal hypoglycemia in 23–26-month-old (aged) mice, which exhibited significantly lower hepatic autophagy than 9-week-old (young) mice. The liver expressions of Bcl-2, an autophagy-negative regulator, and Beclin1–Bcl-2 binding, were increased in aged mice compared with young mice. The autophagy inducer Tat-Beclin1 D11, not the mTOR inhibitor rapamycin, decreased the plasma levels of the glucogenic amino acid and restored the blood glucose levels in aged FR mice. Decreased liver gluconeogenesis, body temperature, physical activity, amino acid metabolism, and hepatic mitochondrial dynamics were observed in the aged FR mice. These changes were restored by treatment with hochuekkito that is a herbal formula containing several autophagy-activating ingredients. Our results indicate that Bcl-2 upregulation in the liver during the aging process disturbs autophagy activation, which increases the vulnerability to undernutrition. The promotion of liver autophagy may offer clinical therapeutic benefits to frail elderly patients.

## Introduction

As the world’s population ages, health care for older people has become a pressing issue. In modern society, many factors, including impaired physical function, psychological disorders, and/or limited social interactions, may result in loss of appetite and/or decreased food intake among the elderly. A pooled analysis of data from older people aged 65 years and over in hospitals in 12 countries reported that 39% were at nutritional risk^1^. Undernutrition leads to frailty, longer hospital stays, frequent readmission, and higher mortality in elderly patients^2–4^. Although nutrition support may be helpful for the management of undernourished patients, cyclic enteral and/or total parenteral nutrition are less effective in elderly patients than in younger individuals^5,6^.

Autophagy is an intracellular degradation system that plays a critical role in supplying nutrients during starvation and likewise performs quality control for intracellular organelles such as the mitochondria^7,8^. Impaired autophagy is closely related to the development of several age-related disorders, including cancer, as well as cardiovascular and neurodegenerative diseases^9^.

Aging results in accumulated damage to molecules, cells, and tissues and ultimately leads to organ malfunction^10^; many organisms show signs of decreased capacity for autophagy that correlates with age^11^, however, the underlying mechanisms are still poorly understood.

The liver is a key metabolic organ that governs energy metabolism^12^ and is capable of amplified metabolic stress-induced autophagy due to lysosome-enriched hepatocytes^13^. Liver autophagy contributes to basic hepatic functions, including gluconeogenesis and β‑oxidation, which fluctuate periodically depending on the fasting–feeding cycle. During prolonged fasting, liver autophagy is critical for maintaining systemic energy homeostasis via gluconeogenesis^14^ and ketogenesis^15^.

We hypothesized that the undernourished elderly experience disrupted energy homeostasis that might be exacerbated by decreased liver autophagy. To explore this possibility, we subjected young and aged mice to severe food restriction (FR) to generate an undernourished condition; we evaluated the distinct metabolic changes and compared the responses of the two groups. Furthermore, we investigated the mechanism controlling liver autophagy and its relationship to energy metabolism during aging.

## Results

### Blood glucose and ketone bodies, body temperature, and spontaneous activity

**Fig. 1:**
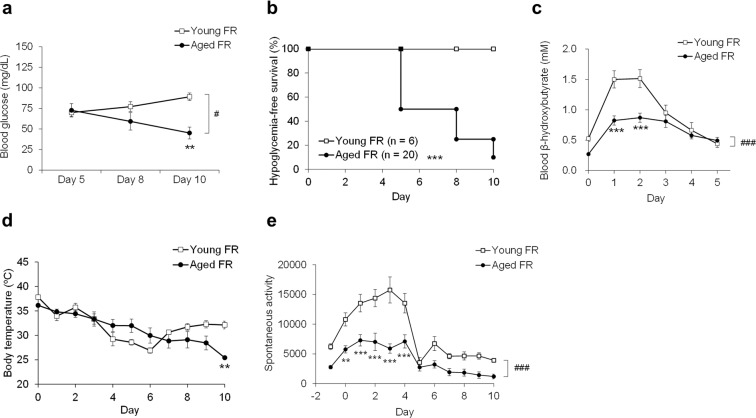
Biochemical and physiological changes on food restriction. **a** Fasting blood glucose levels in young (*n* = 6) and aged mice (*n* = 7–14) on days 5, 8, and 10 after daily food restriction (FR). Two-way ANOVA revealed a significant effect of age (#; *F* (1, 43) = 6.98, *P* = 0.0115) and age × time interaction (*F* (2, 43) = 3.23, *P* = 0.0495); **P < 0.01 vs. young FR mice at this time point by Bonferroni test. **b** Hypoglycemia-free survival; hypoglycemia is defined as blood glucose level <50 mg/dL; ***P < 0.001 vs. young FR mice by log-rank test. **c** Fasting blood β-hydroxybutyrate (βHB) levels in mice on daily FR. Two-way ANOVA revealed significant effects of age (###; *F* (1, 113) = 32.8, *P* < 0.001), time (*F* (5, 113) = 30.6, *P* < 0.001), and the age × time interaction (*F* (5, 113) = 5.87, *P* < 0.001); ***P < 0.001 aged FR mice (*n* = 12–13) vs. young FR mice (*n* = 8) at each time point by Bonferroni test. **d** Body temperature before feeding. Two-way ANOVA revealed significant effects of time (*F* (10, 206) = 10, *P* < 0.001) and age × time interaction (*F* (10, 206) = 2.8, *P* = 0.0029). **P < 0.01 aged FR mice (*n* = 8–20) vs. young FR mice (*n* = 6) at this time point by Bonferroni test. **e** Daily spontaneous activity. Two-way ANOVA revealed significant effects of age (###; *F* (1, 226) = 112, *P* < 0.001), time (*F* (11, 226) = 23.1, *P* < 0.001), and the age × time interaction (*F* (11, 226) = 3.17, *P* < 0.001), **P < 0.01, ***P < 0.001 aged FR mice (*n* = 7–13) vs. young FR mice (*n* = 10) at each time point by Bonferroni test. Data are presented as mean ± SEM (**a**, **c**, **d**, and **e**).

Baseline characteristics of young and aged mice are shown in Supplementary Table 1. There were no significant differences in baseline levels of blood glucose between the two groups. After subjecting the mice to FR, the blood glucose levels were considerably lower than baseline levels on day 5 and had partially recovered in young mice on day 10, but they continued to fall in the aged mice (Fig. 1a). Two-way ANOVA revealed significant effects of age (*P* = 0.0115) and the age × time interaction (*P* = 0.0495). None of the young mice experienced hypoglycemia (defined as blood glucose level <50 mg/dL) at any time during the study period, while hypoglycemia-free survival was significantly diminished among the aged mice (Fig. 1b) accompanied by an increase in mortality (Supplementary Fig. 1a). Transient increases in blood β-hydroxybutyrate (βHB) were observed on days 1 and 2; however, the levels in the aged FR mice were significantly lower than those in the young FR mice (Fig. 1c). Although the body temperature of the young mice subjected to FR initially decreased but ultimately recovered, the decrease in body temperature was persistent and going in the aged FR mice (Fig. 1d). Two-way ANOVA revealed significant effects of time (*P* < 0.001) and also the age × time interaction (*P* = 0.0029). Spontaneous activity initially increased in response to FR but ultimately decreased to less than the baseline activity (i.e., values at day −1) by day 5 in both the young and aged mice; the overall activity was significantly lower in the aged mice than in the young mice (Fig. 1e), but they had a similar percentage change from the baseline (Supplementary Fig. 1b). Two-way ANOVA revealed significant effects of age (*P* < 0.001), time (*P* < 0.001), and the age × time interaction (*P* < 0.001). Taken together, these results suggest that energy homeostasis was disrupted in the aged mice. However, survivorship bias might occur in these data since the 10-day FR resulted in higher mortality in aged mice. Therefore, we limited the duration of FR and the subsequent experiments to 5 days when the survival rate of the aged mice began to decline.

### Body composition and amino acid profile

**Fig. 2:**
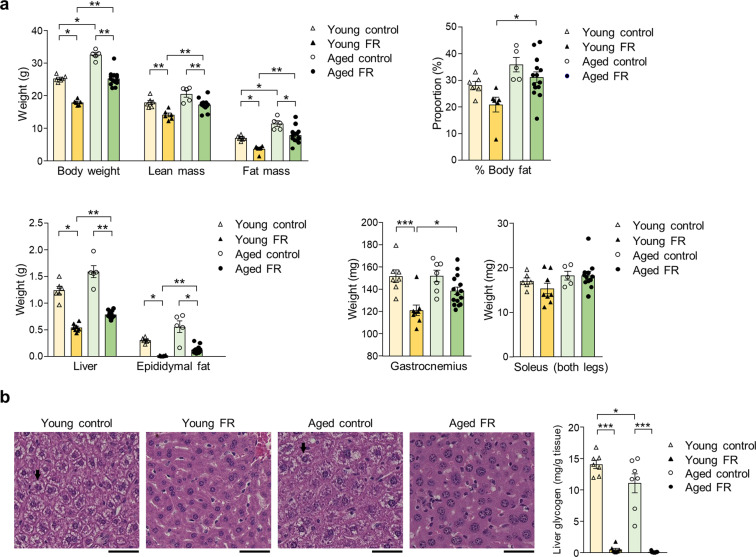
Body composition and liver glycogen on day 5 of food restriction. **a** Body composition in young and aged mice subjected to 5-day food restriction (FR). *P < 0.05, **P < 0.01 by Tukey−Kramer or Steel−Dwass test; young control (*n* = 6), young FR (*n* = 6), aged control (*n* = 5), and aged FR (*n* = 14). Tissue weights in young and aged mice subjected to 5-day FR. *P < 0.05, **P < 0.01, ***P < 0.001 by Tukey−Kramer or Steel−Dwass test. Liver, epididymal fat, and soleus; young control (*n* = 6), young FR (*n* = 8), aged control (*n* = 5), and aged FR (*n* = 12). Gastrocnemius: young control (*n* = 7), young FR (*n* = 8), aged control (*n* = 7), and aged FR (*n* = 15). **b** The hematoxylin and eosin staining of liver sections for liver glycogen content. Arrows indicate sites of glycogen accumulation. Bar = 50 µm. *P < 0.05, ***P < 0.001 by Tukey’s test; young control (*n* = 7), young FR (*n* = 8), aged control (*n* = 7), and aged FR (*n* = 15). Data are presented as mean ± SEM.

**Fig. 3:**
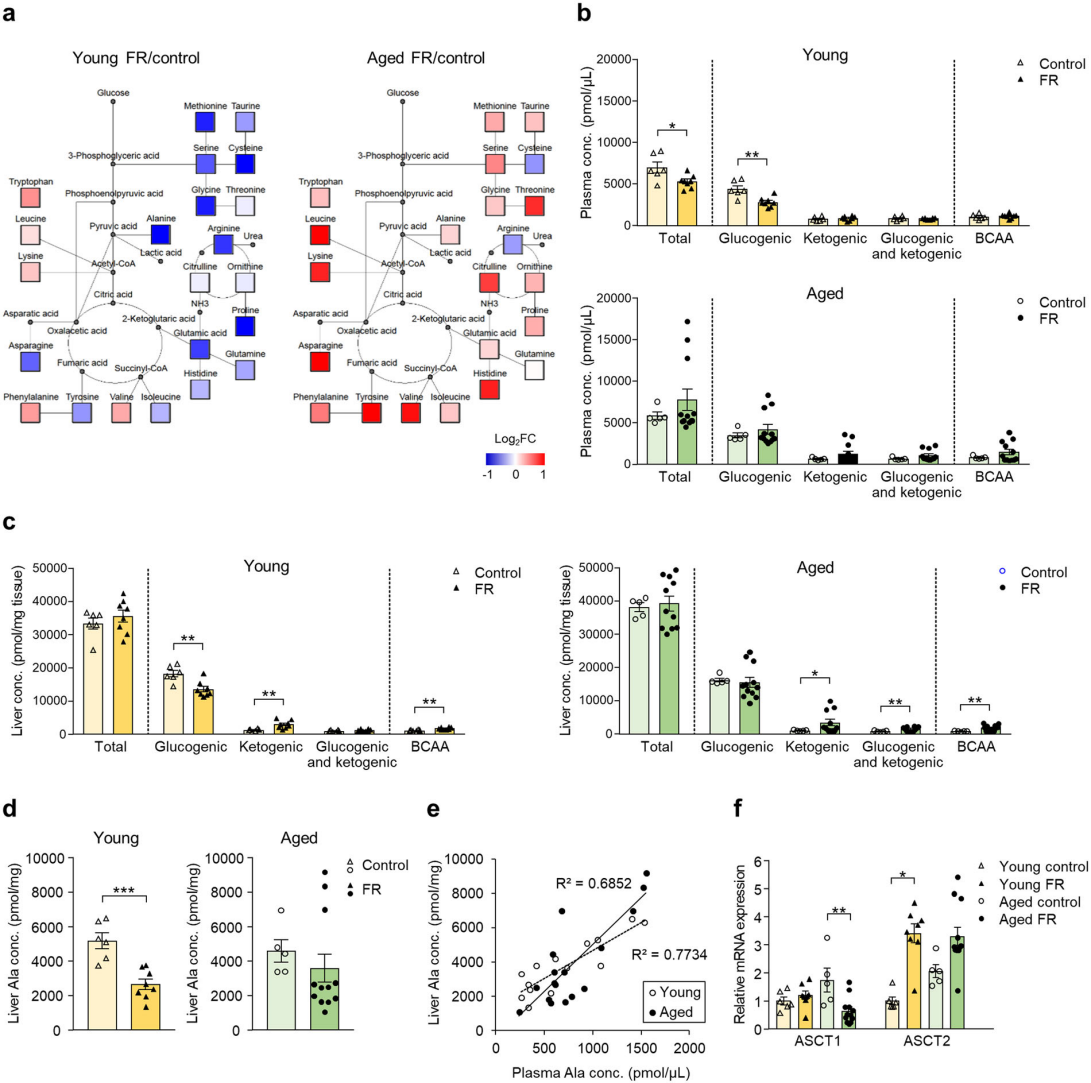
Amino acid profile on day 5 of food restriction. **a** Amino acid metabolic pathway map in young and aged mice all subjected to food restriction (FR). Mean ratio of plasma amino acid levels in FR mice to control mice is shown according to the color scale. Red color represents upregulation and blue color represents downregulation compared to the mean value of age-matched control. Undetected amino acids and key metabolites are shown with small gray circles. **b** Plasma concentration of amino acids. *P < 0.05, **P < 0.01 vs. age-matched controls by Student’s or Aspin–Welch *t* test. **c** The liver concentration of amino acids. *P < 0.05, **P < 0.01 comparison to age-matched controls by the Student’s or Aspin–Welch *t* test. **d** Liver alanine concentration. ***P < 0.001 comparison to the control by the Student’s *t* test. **e** The correlation between liver and plasma alanine concentration in young and aged and control and FR mice. **f** Hepatic gene expression of *Slc1a4*, encoding alanine–serine–cysteine transporter (ASCT) 1, and *Slc1a5*, encoding ASCT2, in the young and aged FR mice. *P < 0.05, **P < 0.01 by the Tukey−Kramer or Steel−Dwass test. Young control (*n* = 6), young FR (*n* = 8), aged control (*n* = 5), and aged FR (*n* = 12). Data are presented as mean ± SEM (**b**, **c**, **d**, and **f**).

We compared the alterations in internal energy sources between the young and aged mice. The body weight of the aged mice was higher than that of the young mice (Fig. 2a). The body composition analysis revealed that the gastrocnemius muscle weight per body weight and the percentage of lean mass were lower in the aged mice than the young mice (Supplementary Fig. 2a, b). Five-day FR resulted in measurable decreases in body weight, lean, and fat mass in both young and aged mice. The percentage of body fat was also significantly higher in the aged FR mice (Fig. 2a). Epididymal fat was depleted in the young mice subjected to FR but could be detected among their aged counterparts (Fig. 2a). FR resulted in a significant reduction in the mass of the gastrocnemius muscle together with a tendency toward reduction of soleus muscle mass in the young mice; interestingly, the decreases observed among the aged mice were smaller and not statistically significant (Fig. 2a). Decreased liver weight (Fig. 2a) and complete depletion of liver glycogen were observed in both the young and aged mice on FR. However, the aged mice had a lower baseline level than the young mice (Fig. 2b), accompanied by distinct changes in plasma amino acid profile secondary to FR (Fig. 3a). Compared with age-matched control mice who were not subjected to FR, total and glucogenic amino acid levels were significantly lower in the plasma of young mice on FR; by contrast, they tended to be higher in the plasma of the aged mice on FR (Fig. 3b).

Similarly, FR decreased the liver levels of glucogenic amino acids in the young mice, but not in the aged mice (Fig. 3c, d). Moreover, a similar positive correlation between the plasma and liver levels of alanine, one of the predominant glucogenic amino acids, was observed both in the young and aged mice (Fig. 3e). These observations suggest that the uptake of alanine into the liver was not decreased in the aged mice, as evidenced by the similar level of expression of Slc1a4, which encodes the alanine–serine–cysteine transporter (ASCT) 1, and Slc1a5, which encodes the ASCT2, in the young and aged FR mice (Fig. 3f). These findings indicate that the aged FR mice may have experienced a reduction in the utilization of amino acids and/or lipids as a fuel source.

### Liver metabolic function

**Fig. 4:**
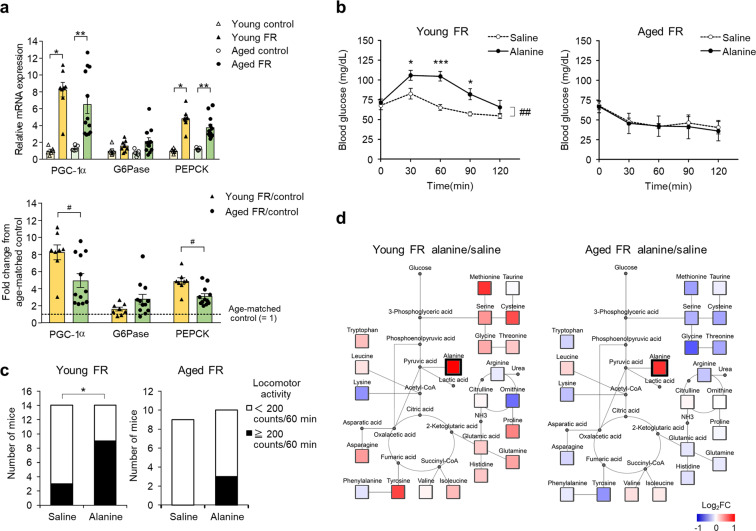
Metabolic differences between young and aged food-restricted mice. **a** Hepatic gene expression in young and aged food-restricted (FR) mice. *P < 0.05, **P < 0.01 by the Steel–Dwass (upper panel) or ^#^*P* < 0.05 by Mann–Whitney (lower panel) test, young control (*n* = 6), young FR (*n* = 8), aged control (*n* = 5), and aged FR (*n* = 12). **b** Changes in blood glucose levels in young (left) and aged (right) FR mice after alanine injection. In the young mice, two-way repeated-measures ANOVA revealed significant effects of the drug (##; *F* (1, 8) = 12.8, *P* = 0.0072), time (*F* (4, 32) = 18.9, *P* < 0.001), and the drug × time interaction (*F* (4, 32) = 4.82, *P* = 0.0037). *P < 0.05, ***P < 0.001 vs. saline-treated mice at each time point by Bonferroni test; *n* = 5 mice. In the aged mice, two-way repeated measures of ANOVA revealed the significant effect of time (*F* (4, 24) = 8.92, *P* < 0.001), *n* = 4. **c** The proportions of mice divided by the degree of locomotor activity between 60 and120 min after alanine injection. *P < 0.05 by chi-square test; young FR saline (*n* = 14), young FR alanine (*n* = 14), aged FR saline (*n* = 9), and aged FR alanine (*n* = 10). **d** Amino acid metabolic pathway map in young and aged mice subjected to FR at 180 min after alanine injection. Mean ratio of plasma amino acid levels detected in alanine-treated mice to saline-treated mice is shown according to the color scale. Red color represents upregulation and blue color represents downregulation compared to the mean values of age-matched saline-treated mice. Undetected amino acids and key metabolites are shown with small gray circles. Data are presented as mean ± SEM (**a**, **b**).

Our results thus far suggest that hypoglycemia observed in the aged FR mice might be attributable to impaired gluconeogenesis. As such, we examined the expression of gluconeogenesis-related genes in the liver, the organ that provides critical regulation of glucose homeostasis. There were no differences in baseline expression of peroxisome proliferator-activated receptor-γ coactivator (PGC)-1α, glucose 6-phosphatase (G6Pase), or phosphoenolpyruvate carboxykinase (PEPCK) in liver tissues of the young and aged mice (Fig. 4a). Expression of these metabolic factors increased by day 5 of FR; however, the increase in PGC-1α and PEPCK expression in aged mice was significantly lower than in young mice (Fig. 4a). Next, an alanine tolerance test was conducted to assess the hepatic capacity for gluconeogenesis, amino acid metabolism, and activation of locomotor activity. There was a marked increase in blood glucose levels after alanine injection in the young mice on FR (Fig. 4b). Two-way repeated-measures ANOVA revealed significant effects of the drug (*P* = 0.0072), time (*P* < 0.001), and the drug × time interaction (*P* = 0.0037). Aged mice on FR with blood glucose levels that were similar to those of young mice on FR before alanine injection (i.e., <100 mg/dL) were selected for the alanine tolerance test so that we would be able to perform a rigorous comparison of the capacity for gluconeogenesis. Interestingly, blood glucose levels in the aged FR mice did not increase at all after the alanine injection (Fig. 4b); these results suggest a decreased capacity for gluconeogenesis among the aged mice. A significantly larger number of younger FR mice who received alanine injections exhibited more than 200 counts/60 min of locomotor activity compared to the younger mice treated with saline; by contrast, no significant differences were observed when comparing alanine vs. saline injection in the aged FR mice (Fig. 4c). To assess amino acids' metabolic changes among all mice subjected to FR, we evaluated the results of a plasma amino acid analysis performed 180 min after the alanine injection. Ratios of amino acid levels in the alanine-injected group vs. the saline-injected group are shown by color scale in Fig. 4d. Among the younger FR mice, most amino acids increased in response to the alanine injection; no changes were observed in response to alanine injection among the aged FR mice.

### Autophagy

**Fig. 5:**
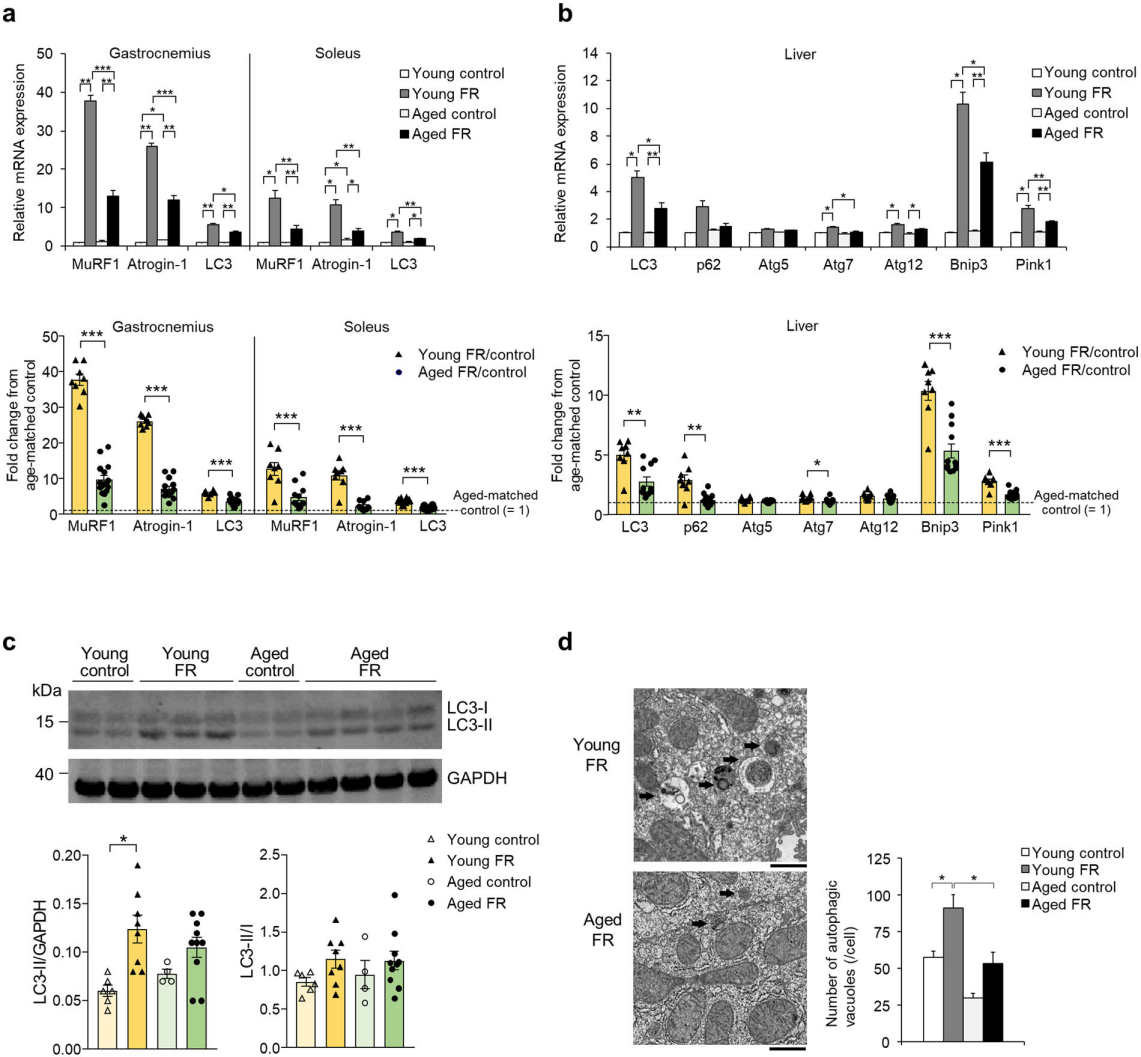
Proteolysis and autophagy in muscle and liver on day 5 of food restriction. The gene expression in **a** muscle and **b** liver of young and aged food-restricted (FR) mice, *P < 0.05, **P < 0.01, ***P < 0.001 by the Steel–Dwass test (upper panel) or Student’s or Aspin–Welch *t* test (lower panel). Gastrocnemius: young control (*n* = 7), young FR (*n* = 8), aged control (*n* = 7), and aged FR (*n* = 15). Soleus and liver: young control (*n* = 6), young FR (*n* = 8), aged control (*n* = 5), and aged FR (*n* = 12). **c** LC3-II protein expression levels and LC3-II/I ratio in the liver with representative western blot images. Blots were cropped to improve conciseness, and full-length blots are presented in Supplementary Fig. 5. *P < 0.05 by Steel–Dwass test: young control (*n* = 6), young FR (*n* = 8), aged control (*n* = 4), and aged FR (*n* = 10). **d** Electron microscopic images of hepatic autophagic vacuoles and the number per cell (five hepatocytes/mice, *n* = 3 mice/group). Bar = 1 μm. Arrows indicate autophagic vacuoles, *P < 0.05 by Tukey−Kramer test. Data are presented as mean ± SEM.

We examined two major proteolysis pathways in the two mouse cohorts, the ubiquitin–proteasome system and autophagy. Expression of E3 ubiquitin ligases (MuRF1 and Atrogin-1), and autophagy-related protein LC3 in both gastrocnemius and soleus muscles, was increased by 5 day of FR; this increase was significantly attenuated in the aged FR mice compared with the young FR mice (Fig. 5a). There were no significant differences in the baseline levels of these proteins between young and aged mice, except for Atrogin-1, which was significantly higher in the aged mice at baseline than in the younger mice (Fig. 5a). Hepatic expression of autophagy- and mitophagy (selective mitochondrial autophagy)-related genes, including adenovirus E1B 19-kDa-interacting protein 3 (Bnip3) and PTEN-induced kinase 1 (Pink1), increased in response to FR; this increase was attenuated in the aged FR mice (Fig. 5b). Likewise, expression of autophagic marker LC3-II protein was significantly increased in response to FR in the young mice but not in the aged mice (Fig. 5c). Baseline levels of these gene expressions were nearly comparable in both young and aged mice (Fig. 5b).

Electron microscopic analysis of liver tissue revealed that the number of autophagic vacuoles within the hepatocytes increased in response to FR in both the young mice but not in the aged mice, and they were significantly lower in the aged FR mice than in the young FR mice (Fig. 5d). Interestingly, the morphological characteristics of the hepatocyte mitochondria were somewhat different when comparing those from young to aged mice (Supplementary Fig. 3a). In the hepatocytes from young mice subjected to FR, mitochondria were enlarged with highly stacked cristae; by contrast, those in the aged mice subjected to FR appeared to be swollen with sparse cristae. Expression of mitochondrial fusion and fission genes was increased in response to FR, but the increase was attenuated in the aged mice compared to that in young mice (Supplementary Fig. 3b). Taken together, these findings suggest impaired induction of hepatic autophagy and mitophagy in aged mice subjected to FR.

### The association of autophagy and gluconeogenesis and the interaction between Beclin 1 and Bcl-2

**Fig. 6:**
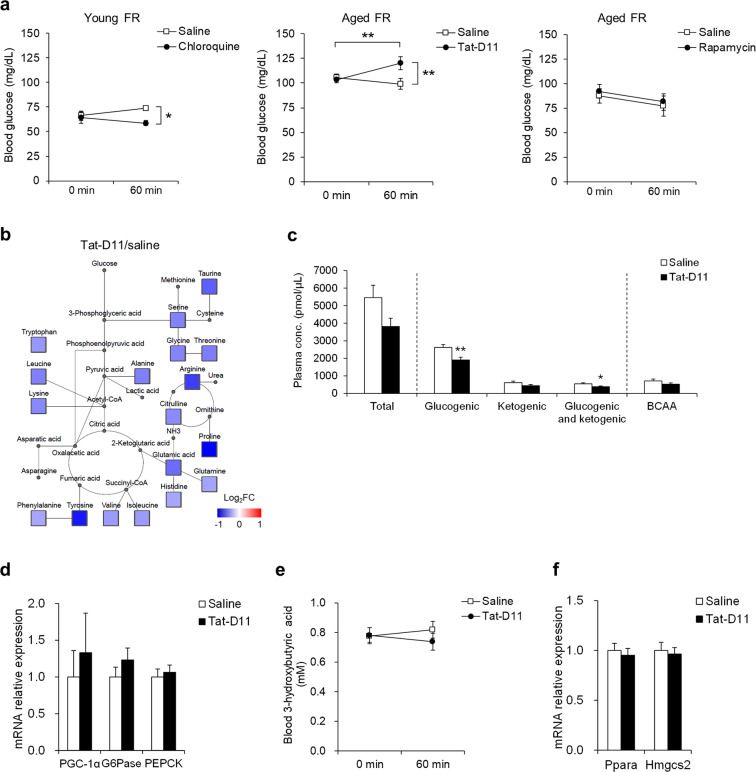
Effect of autophagy modulators on day 5 of food restriction. **a** Blood glucose levels after injection of chloroquine in young food-restricted (FR) mice (left panel; saline *n* = 4, chloroquine *n* = 5), after injection of Tat-Beclin1 D11 autophagy-inducing peptide (Tat-D11) in aged FR mice (middle panel; saline *n* = 17, Tat-D11 *n* = 16), and rapamycin in aged FR mice (right panel; saline *n* = 11, rapamycin *n* = 10). *P < 0.05, ***P < 0.001 by Bonferroni test. **b** Map of an amino acid metabolic pathway in aged FR mice at 60 min after Tat-D11 injection. Mean ratio of the plasma level of amino acids in Tat-D11-injected mice to that in saline-injected mice was shown in the color scale, where red and blue represented upregulation and downregulation compared to the mean value in saline-injected mice, respectively. Undetected amino acids and key metabolites are denoted with small gray circles. **c** The plasma concentration of the amino acids. **d** The gene expression of gluconeogenic-related peptides in the liver. **e** Blood βHB levels. **f** Gene expression of ketogenesis-related peptides in the liver 60 min after Tat-D11 injection in aged FR mice. *P < 0.05, **P < 0.01 Comparison to the saline-injected mice by the Student’s *t* test; saline (*n* = 17), Tat-D11 (*n* = 16). Data are presented as mean ± SEM.

**Fig. 7:**
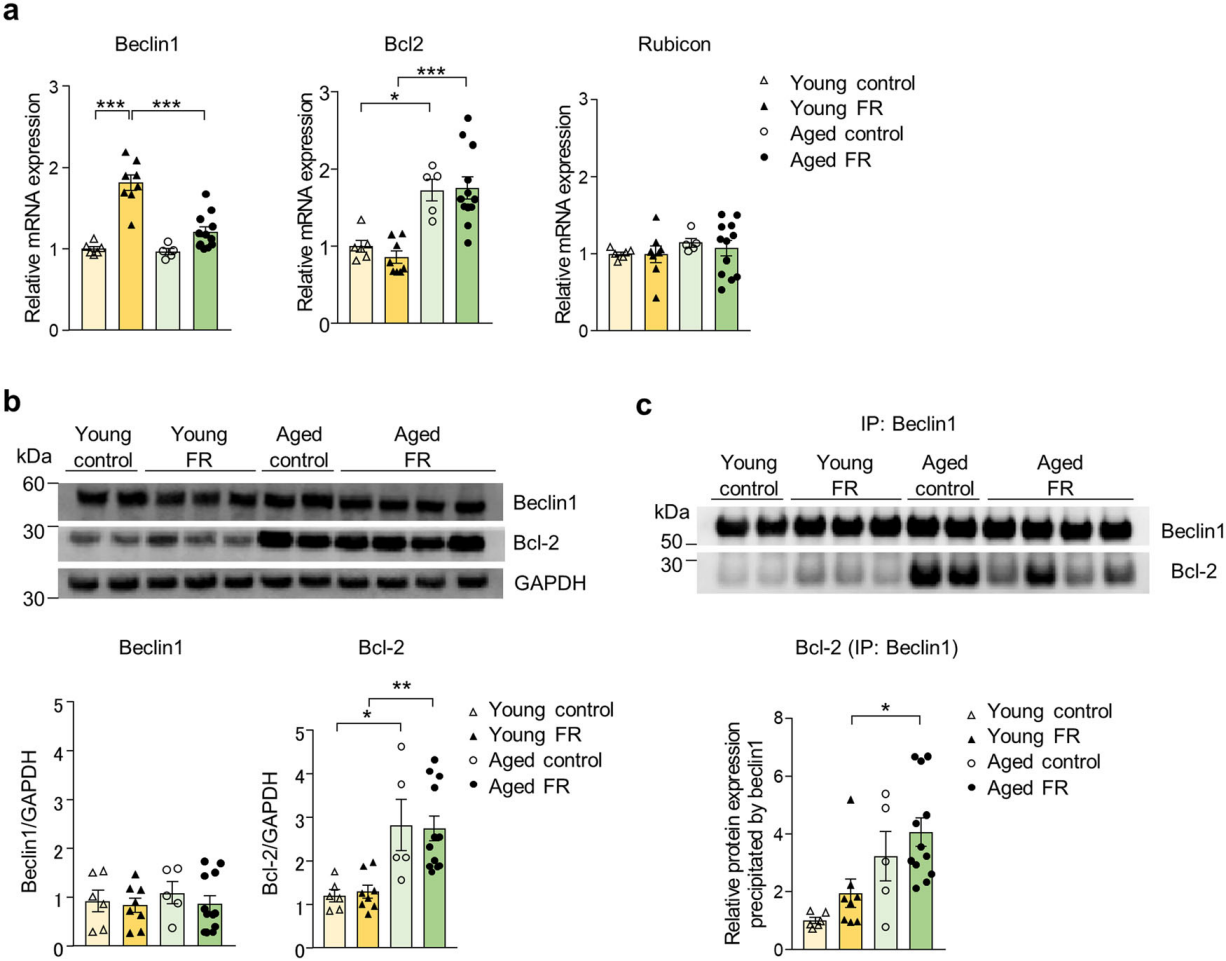
Interaction between Beclin 1 and Bcl-2 on day 5 of food restriction. **a** Relative mRNA expression levels of autophagy-related peptides in the liver. Protein expression levels of beclin1 and Bcl-2 in the liver with representative (**b**) western blot images and **c** co-immunoprecipitation analysis. The shown blots were cropped to improve the conciseness and the full-length blots are presented in Supplementary Fig. 5. *P < 0.05, **P < 0.01, ***P < 0.001 by Tukey test: young control (*n* = 6), young food-restricted (FR) (*n* = 8), aged control (*n* = 5), and aged FR (*n* = 12). Data are presented as mean ± SEM.

To investigate the association of autophagy with gluconeogenesis, we examined the impact of administration of the autophagy inhibitor, chloroquine, in the young mice on FR and the autophagy inducers, Tat-D11, and rapamycin, in their aged counterparts. Blood glucose levels after chloroquine injection were significantly lower than those detected after saline injection among the young FR mice (Fig. 6a). Among the aged FR mice, Tat-D11, but not rapamycin, resulted in an increase in blood glucose levels (Fig. 6a). The plasma levels of the amino acids, especially glucogenic, were significantly decreased by Tat-D11 injection (Fig. 6b, c). However, the hepatic expression of genes encoding the gluconeogenic enzymes was similar in the Tat-D11- and saline-treated aged mice (Fig. 6d). In addition, there was no change in the blood βHB levels (Fig. 6e), expression of *Pparα*, encoding the fatty acid oxidation-related gene peroxisome proliferator-activated receptor α (PPARα) and *Hmgcs2*, encoding the critical enzyme ketogenesis 3-hydroxy-3-methylglutaryl coenzyme A synthase 2 (HMGCS2) (Fig. 6f). To clarify the mechanism of impaired autophagy in the aged FR mice, we analyzed the expression of autophagy-regulating proteins beclin1 and Bcl-2. Expression of beclin1 was significantly increased in response to FR among the young mice; the parallel increase in the aged mice was not significant (Fig. 7a). Expression of Bcl-2 was significantly higher in the aged mice than in the young mice, regardless of FR (Fig. 7a). There were no significant differences in the expression of Rubicon in any of the mice evaluated (Fig. 7a). Expression of immunoreactive Beclin1 was comparable among all groups evaluated, while expression of immunoreactive Bcl-2 was significantly higher in the aged mice, regardless of FR (Fig. 7b). An immunoprecipitation assay revealed stronger binding between Beclin1 and Bcl-2 in the aged FR mice than in the young FR mice (Fig. 7c).

### Autophagy induction and regulation of energy homeostasis by hochuekkito (HET) treatment

Next, we investigated the effects of HET on energy homeostasis in aged FR mice, because recent studies have demonstrated the potential of HET to induce autophagy^16,17^ and restore metabolic homeostasis^18^. The results in Table 1 demonstrate the capacity of HET and its ingredients to induce autophagy as assessed by HEK293 autophagy-reporter cells. HET at 500 μg/mL induced autophagy more effectively than vehicle control alone (*P* < 0.001 vs. vehicle-treated wells). Among 66 chemical ingredients in 10 components of HET tested, 9 ingredients had the capacity to induce autophagy in the in vitro assay system.

**Table 1:** Autophagy induction (% of control) by hochuekkito (HET) and its active ingredients in vitro.

Test substance	Low concentration			High concentration		
	Concentration	Induction (%)	*P* value	Concentration	Induction (%)	*P* value
HET	100 μg/mL	103.5 ± 5.1	0.9208	500 μg/mL	133.5 ± 3.2	<0.001
Calycosin (astragalus root)	5 μmol/L	163.6 ± 6.3	<0.001	25 μmol/L	188.7 ± 0.4	<0.001
Formononetin (astragalus root)	5 μmol/L	113.5 ± 3.9	0.1118	25 μmol/L	142.9 ± 3.3	<0.001
6-gingerol (ginger)	5 μmol/L	108.3 ± 4.8	0.3762	25 μmol/L	136.1 ± 3.3	<0.001
8-gingerol (ginger)	5 μmol/L	109.4 ± 4.6	0.2816	25 μmol/L	162.8 ± 1.4	<0.001
Oleanolic acid (jujube)	5 μmol/L	116.2 ± 1.7	<0.001	25 μmol/L	130.8 ± 0.2	<0.001
Betulonic acid (jujube)	5 μmol/L	157.4 ± 3.1	<0.001	25 μmol/L	146.8 ± 2.2	<0.01^a^
Oleanonic acid (jujube)	5 μmol/L	116.2 ± 11.0	0.2609	25 μmol/L	165.3 ± 1.6	<0.001
Maslinic acid (jujube)	5 μmol/L	138.7 ± 0.9	<0.001	25 μmol/L	133.3 ± 1.2	<0.001^a^
Compound K (ginseng)	5 μmol/L	105.1 ± 4.5	0.6153	25 μmol/L	145.2 ± 0.7	<0.001

Data are shown as the mean ± SEM (*n* = 3). *P* values versus vehicle-treated wells were determined by the Dunnett test.

^a^Cell toxicity was observed.

**Fig. 8:**
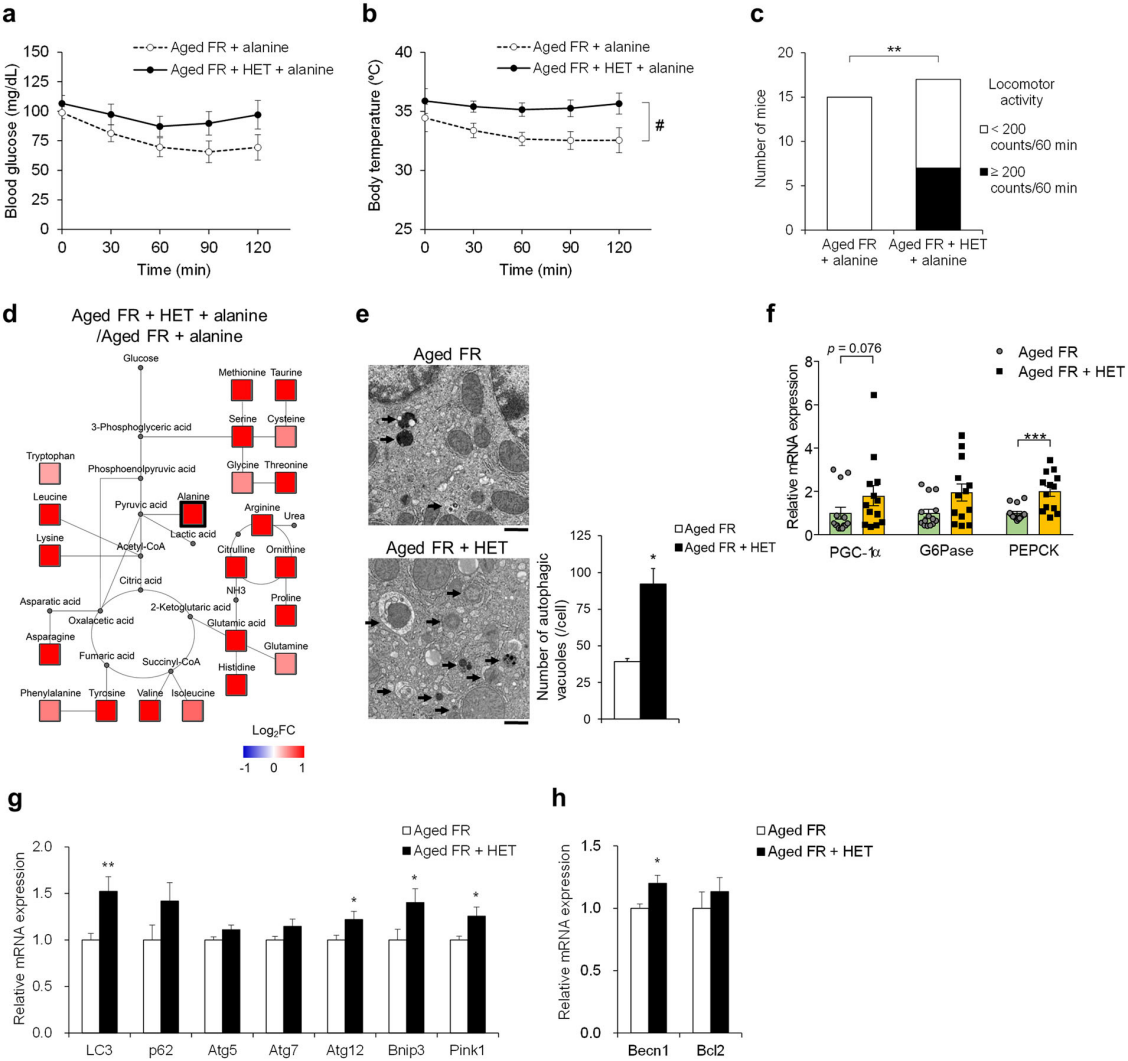
Effect of hochuekkito on metabolic function on 5-day food restriction. Changes in **a** blood glucose and **b** body temperature after alanine injection in aged food-restricted (FR) mice receiving hochuekkito (HET)-containing diet (*n* = 11) or control diet (*n* = 14). With respect to body temperature, two-way repeated-measures ANOVA revealed significant effects of HET (#; *F* (1, 23) = 5, *P* = 0.0354) and time (*F* (4, 92) = 2.86, *P* = 0.0275). **c** Mice were divided by levels of locomotor activity at 60 min to 120 min after alanine injection, **P < 0.01 by chi-square test; control (*n* = 15), HET (*n* = 17). **d** Amino acid metabolic pathway map determined from levels detected at 180 min after alanine injection. Mean ratio of plasma amino acid levels in aged FR mice who received HET-containing diet to control diet was shown according to the color scale. Red color represents upregulation and blue color represents downregulation compared to the mean value of mice receiving control diet. **e** Electron microscopic images of hepatic autophagic vacuoles and the number per cell (five hepatocytes/mice, *n* = 4 mice/group). Bar = 3 μm. Arrows indicate autophagic vacuoles, ***P < 0.001 vs. aged FR by Aspin–Welch *t* test. **f** Relative mRNA expression levels of gluconeogenic-related proteins in the liver, ***P < 0.001 by Mann–Whitney test; control (*n* = 14), HET (*n* = 13). **g**, **h** Relative mRNA expression levels of autophagy-related peptides in the liver, *P < 0.05, **P < 0.01 vs. aged FR by Student’s or Aspin–Welch *t* test; control (*n* = 14), HET (*n* = 13). Data are presented as mean ± SEM (**a**, **b**, **e**, **f**, **g**, and **h**).

We then proceeded to examine the impact of HET on hepatic metabolic function and autophagy in aged mice. There were no significant differences in the body weight, adipose tissue weight, or other parameters between the 4-week HET-treated and control aged mice (Supplementary Table 2). On day 5 of FR, HET treatment had no significant impact on blood glucose levels and body temperature in aged mice (values at time 0 in Fig. 8a, b). After alanine injection, the blood glucose levels in the aged FR mice who received the control diet were significantly lower than those before injection (120 min, 69.4 vs. 0 min, 98.9 mg/dL, *P* < 0.001). This was not observed in the aged FR mice who received HET-containing diet, and the blood glucose levels tended to be higher than those measured in the aged FR mice who received control diet (Fig. 8a). Body temperature after the alanine injection was significantly higher in the HET-treated mice than in control mice (Fig. 8b). Two-way repeated-measures ANOVA revealed significant effects of the drug (*P* = 0.0354) and time (*P* = 0.0275). The number of mice that exhibited more than 200 counts/60 min of locomotor activity after alanine injection was significantly higher among the HET-treated aged FR mice than in their control counterparts (Fig. 8c). The level of plasma amino acids after alanine injection was overall high in response to HET treatment among aged FR mice (Fig. 8d). HET treatment significantly increased the number of autophagic vacuoles relative to those enumerated in hepatocytes from control counterparts (Fig. 8e). We assessed the change in hepatic gene expressions in response to FR; HET-treated aged mice exhibited significant high expression levels in gluconeogenesis-related genes (Fig. 8f), autophagy- and mitophagy-related genes (Fig. 8g, h), and mitochondrial fusion and fission genes (Supplementary Fig. 4a), compared with control counterparts.

HET is reported to have a potential clinical anti-inflammatory effect^19,20^. A recent study has demonstrated the association between the decline in autophagy and age-related inflammation^21^. Therefore, we assessed the expression of inflammation-related genes in the liver to determine whether HET exerted an anti-inflammatory effect. The inflammatory parameters were increased in aged mice, but the suppression by the 4-week treatment of HET was insignificant (Supplementary Fig. 4b).

## Discussion

This study demonstrated that the aged mice experienced fatal hypoglycemia in association with impaired gluconeogenesis during severe FR; these findings were accompanied by decreased liver metabolic capacity and physical activity. We also found that activation of hepatic autophagy was decreased in aged FR mice compared to young FR mice. Specifically, the interaction of Beclin1, an essential protein for autophagy, with its negative regulator Bcl-2, was increased due to high levels of Bcl-2 expression in the aged FR mice. The autophagy inducer Tat-Beclin1 D11 restored gluconeogenesis accompanied by the decreased plasma level of glucogenic amino acids, and HET that contains autophagy-activating ingredients improved gluconeogenesis, amino acid metabolism, body temperature, and locomotor activity in the aged FR mice.

Inadequate nutrition in the elderly leads to frailty and associated high morbidity and mortality^2–4^. In this study, severe FR on aged mice induced persistent hypoglycemia with hypothermia. Interestingly, young mice on FR recovered from decreased blood glucose and body temperature after day 5 until day 10. These results demonstrate that the aged mice were highly vulnerable to the physiological metabolic stress caused by dietary restriction compared with young mice. This novel and highly clinically relevant model will be very useful to others interested in exploring the metabolic insults associated with aging.

When faced with starvation, various biological adaptations are made in order to maintain energy homeostasis. For example, locomotor activity usually increases during the early phase of fasting or severe food restriction in mice, a response known as food foraging behavior^22^. This behavior was attenuated in the aged mice compared with the young mice in this study; these results suggest a focus on decreased energy consumption in the aged mice. In regard to energy production, glucose and ketone bodies produced in the liver play key roles in providing essential energy for extrahepatic tissues during fasting and starvation^12^. In the fasted state, glycogen is degraded to supply glucose, and fatty acids released from adipose tissue are oxidized to generate ketone bodies (ketogenesis); furthermore, gluconeogenic substrates, such as lactate, glycerol, and amino acids, are converted to glucose via the process known as gluconeogenesis. In this study, glycogen stores in the liver were depleted both in the young and aged mice at day 5 of FR. Blood βHB increased in the early phase of FR but returned to the basal levels on day 5 in the young mice who had already exhausted their fat stores and had the little additional capacity to produce ketone bodies. In the aged mice, the increase in blood βHB levels was attenuated relative to that detected in the young mice, and weight and proportion of body fat on day 5 of FR were higher in the aged mice than among their younger counterparts. As such, we assumed that the capacity for ketogenesis and/or lipolysis was diminished in the aged FR mice. Liver glycogen shortage during fasting was reported to facilitate lipolysis in adipose tissues^23^. Since aged mice have a lower baseline of glycogen content, liver glycogen depletion may occur more quickly in them. Thus, the reduction of ketogenesis or lipolysis or both in the aged FR mice may not be related to the liver glycogen levels; further study is needed to clarify this issue. In the young mice subjected to a 5-day FR, muscle weights decreased but blood glucose was maintained >50 mg/dL; these responses were likewise attenuated in the aged FR mice, many of whom became markedly hypoglycemic. These findings indicate that the supply of amino acids due to proteolysis could be insufficient in the aged FR mice. While gluconeogenesis accompanied by accelerated proteolysis was observed among rats fasted for 4 days, plasma levels of amino acids such as alanine decreased with time^24^. In our study, total and glucogenic amino acids in plasma were decreased in the young FR mice, but were paradoxically increased in their aged counterparts. The relationship between the levels of glucogenic amino acid in the plasma and liver and the comparable expression levels of genes encoding the transporters is similar in the young and aged FR mice. Therefore, amino acids, especially glucogenic amino acids such as alanine, are taken up into the liver in both young and aged mice. On the other hand, alanine is converted into pyruvate in the liver and transported into the mitochondrial matrix via the mitochondrial pyruvate carrier (MPC, an MPC1 and MPC2 protein complex) for gluconeogenesis during fasting. The expression of *Mpc2*, encoding MPC2, was decreased in the aged FR mice compared to the young FR mice (Supplementary Fig. 2c). These data imply that the decrease in pyruvate uptake into mitochondria may be partially associated with impaired gluconeogenesis in the aged mice. Taken together, these results suggest that aged mice have only a limited capacity to utilize amino acids for energy in the setting of FR.

As such, we evaluated the capacity for alanine-induced gluconeogenesis as an index for amino acid usage. As a result, unlike young FR mice, this pathway was unavailable to the aged FR mice. In addition, FR induced an increase in hepatic expression of cytosolic PEPCK, an enzyme that serves as a rate-limiting step in gluconeogenesis^25^, and PGC-1α, a key modulator of gluconeogenesis^26^; increase in these mRNA expressions by FR was significantly attenuated in aged FR mice, suggesting a significant deterioration of the gluconeogenic capacity. Moreover, the increase in plasma levels of various amino acids and locomotor activity after alanine injection observed in young FR mice were not in evidence among the aged FR mice, indicating a disruption in amino acid metabolism. These findings suggest that the hepatic systems in place that respond to undernutrition were impaired in the aged mice; the lack of these protective mechanisms is likely to be responsible for the high mortality rates observed.

A previous publication reported that a decrease in plasma glucose that ensued during 24 h of starvation was augmented in mice devoid of the liver-specific autophagy genes^14^; these results indicate that liver autophagy may be associated with the maintenance of blood glucose. In this study, we showed that treatment with chloroquine, which has been reported to suppress autophagy^27^, reduced blood glucose levels in the young FR mice; these results underscored the importance of autophagy in maintaining of blood glucose levels as well. Recent reports have demonstrated an association between aging and dysfunctional autophagy^9,11^. Here, we investigated liver autophagic responses to undernutrition in aged mice and found that induction of autophagy was attenuated in this cohort; these findings were based on morphological observations together with protein and genetic analysis. The results suggest that decreased induction of autophagy might be involved in promoting the sustained hypoglycemia that is a characteristic of the aged FR mice. Mitochondria, which are organelles that modulate energy production, can undergo fusion in order to proceed with efficient energy production and to escape from autophagic degradation in the fasting state^28^. In our study, the mitochondria exhibit distinct morphological characteristics in response to FR in both young and aged mice, including fusion-like morphology in hepatocytes from young FR mice and swollen microarchitecture in hepatocytes from the aged FR mice. We hypothesize that these aberrancies are related to dysfunctional autophagy in the aged mice, since mitochondria-specific autophagy is involved primarily in mitochondrial quality control; however, further exploration will be required in order to identify the pathways involved in these responses.

Although various factors are capable of regulating autophagy, mTOR is a major factor involved in regulating the induction of autophagy as a nutrient sensor^29^. Interestingly, rapamycin, which is a specific mTOR inhibitor, had no impact on the blood glucose levels in the aged FR mice. By contrast, Beclin1 is a protein required at the early stages of autophagosome formation^30^. Tat-D11, which can induce autophagy by releasing endogenous beclin1 from negative regulators^31^, served to increase blood glucose levels in the aged FR mice. These results indicate that the Beclin1-related autophagy pathway located downstream of mTOR might be involved in impaired glucose homeostasis in the aged FR mice. Remarkably, the Tat-D11 treatment decreased the plasma level of the glucogenic amino acids without affecting ketogenesis or changing the expression of the genes encoding the gluconeogenic enzymes. As mentioned above, aged mice had a limited capacity to utilize amino acids for energy under FR conditions. These results indicate that autophagy activation enhanced gluconeogenesis by promoting glucogenic amino acid utilization in undernourished aged mice without activating the gluconeogenic enzymes. Further investigations are required to clarify its underlying mechanism.

Bcl-2^32^ and Rubicon^33^ both inhibit Beclin1-mediated autophagy, and 48-h fasting-induced autophagy was reduced in Bcl-2-overexpressing mice^32^. The results from another study revealed an increase in autophagy and an increased lifespan of mice that expressed Beclin1 with a mutation that did not permit interactions with Bcl-2^34^. In this study, the increase in Beclin1 expression due to FR was attenuated in the aged mice. Moreover, we identified the aging-associated expression of Bcl-2 in the whole liver; the Beclin1–Bcl-2 interaction also increased significantly with aging. These findings suggest that the increase in Bcl-2 protein in the aged mice promotes interactions with Beclin1 and thereby inhibits its activity, leading to suppression of autophagosome formation; this aberrant pathway may contribute to the limited extent of autophagy that can be induced by FR in the aged mice. Bcl-2 is localized not only on the outer mitochondrial membrane but also on the endoplasmic reticulum (ER) membrane; autophagy is inhibited by the interaction between Beclin1 and Bcl-2 only when they are located on ER, not on the mitochondria^32^. Several studies directed at revealing mechanisms of apoptosis reported that expression of Bcl-2 protein on the liver mitochondrial membrane was reduced among aged rats^35,36^. As Bcl-2 expression in total liver homogenates was increased in the aged mice cohort in our study, Bcl-2 localization on the ER membrane or in the cytoplasm might increase with aging, although the detailed mechanism remains unclear. Expression of Rubicon, a protein that inhibits autophagy by binding to Beclin1 at the autophagosome maturation step, did not increase in response to aging in the present study. These results suggest that Beclin1-mediated autophagy was inhibited not by Rubicon but by Bcl-2.

HET is one of Kampo medicines, which is prescribed for patients after surgery or as treatment for illnesses associated with general fatigue or poor appetite. Clinical studies have reported that HET improves fatigue, lack of energy, and low body temperature in adult patients with appetite loss^37^, QOL and immunological status of elderly patients with weakness^38^, and nutritional status in elderly patients with chronic obstructive pulmonary disease^39^. Several studies carried out in vitro have demonstrated the potential of HET to restore metabolic homeostasis^18^ and promote autophagy^16,17^. We confirmed that HET and 9 of its chemical ingredients derived from astragalus root, jujube, ginger, and ginseng induced autophagy in studies featuring reporter cells; these studies suggest that these compounds may contribute to the inductive effects of HET. Among them, 6 components have been identified as capable of inducing autophagy in vitro^40–45^. To the best of our knowledge, this is the first study to identify induction of autophagy by 8-gingerol (ginger-derived), oleanonic acid, and betunolic acid (jujube-derived).

We investigated the impact of HET on energy homeostasis in aged FR mice. Chronic administration of HET facilitated the partial recovery of energy production as indicated by the increased body temperature, locomotor activity, and blood glucose after alanine injection in the aged FR mice treated with HET; these findings were accompanied by increased expression of gluconeogenic enzymes in the liver. Moreover, HET treatment resulted in an increase in plasma levels of various amino acids after alanine injection in the aged FR mice; these findings suggested that HET could restore the impaired hepatic energy metabolism in aged mice. Furthermore, we confirmed that HET was capable of inducing liver autophagy in the aged FR mice as assessed by the increased number of autophagic vacuoles and significantly increased expression of autophagy-related genes. Although the detailed mechanism underlying the responses to HET is unclear, compounds that increase Beclin1 expression^40^ and block the Beclin1–Bcl-2 interaction^43^ may contribute to its mechanism of action. Therefore, HET could alleviate the disruption of energy homeostasis during the aging process by mainly inducing liver autophagy, but not anti-inflammatory effects. HET may facilitate the recovery from decreased physical strength in older patients with undernutrition.

Renal gluconeogenesis is reported to produce 15–25% of the circulating glucose in the postabsorptive state; this fraction increases as starvation progresses^46^. Further studies will be needed so that we have a clearer understanding of autophagy in the kidney. Moreover, although we demonstrated restoration of energy homeostasis in response to HET in the aged mice, further investigation will be needed to determine if HET has any impact on autophagy and the capacity to generate energy among elderly people.

In summary, this study revealed that decreased activation of liver autophagy secondary to increased expression of Bcl-2 and enhancement of the Beclin1–Bcl-2 interaction might lead to the disrupted energy production and contribute to the vulnerability to undernutrition among aged mice. Improvement in liver autophagy is critical for maintaining energy homeostasis in the elderly; this process could be an important therapeutic target for combating frailty and related disorders with poor prognoses.

## Methods

### Animals

Male C57BL/6J mice (Charles River Laboratories, Tokyo, Japan) aged 9 weeks (young) and 23–26 months (aged) were used in our experiments. The mice were housed singly in a controlled environment (20–26 °C, 30–70% humidity) under a 12-h light/dark cycle with free access to standard powdered chow and water. This study was approved by and was conducted according to the guidelines of the experimental animal ethics committees of Tsumura & Co. (Tokyo, Japan, permit no.15-037, 15-074, 16-069, 17-003, 17-036), and the animal experiments were conducted in compliance with the ARRIVE guidelines.

### Test substances

l-Alanine (Wako, Osaka, Japan), chloroquine (Sigma-Aldrich, St. Louis, MO), and Tat-Beclin1 D11 autophagy-inducing peptide (Tat-D11, Novus Biologicals, Centennial, CO) were dissolved in saline. Rapamycin (LC Laboratories, Woburn, MA) was dissolved in saline containing 0.001% dimethyl sulfoxide. The Kampo medicine HET is a dried and powdered hot-water extract of the following ten components: Astragalus Root (*Astragali radix*) 4.0 g, Atractylodes Lancea Rhizome (*Atractylodis lanceae rhizoma*) 4.0 g, Ginseng (*Ginseng radix*) 4.0 g, Japanese Angelica root (*Angelicae radix*) 3.0g, Bupleurum Root (*Bupleuri radix*) 2.0 g, Jujube (*Zizyphi fructus*) 2.0 g, Citrus Unshiu Peel (*Aurantii nobilis pericarpium*) 2.0 g, Glycyrrhiza (*Glycyrrhizae radix*) 1.5 g, Cimicifuga Rhizome (*Cimicifugae rhizoma*) 1.0 g, and Ginger (*Zingiberis rhizoma*) 0.5 g. HET was manufactured by Tsumura & Co. (Tokyo, Japan) and used by mixing in standard chow.

### Food restriction

Young and aged mice were placed on a 70% restricted diet, which provided 30% of their daily food intake at 1.1 and 1.3 g/day, respectively, as measured during the acclimatization period. Day 0 was defined as the first day of FR. These mice underwent the 70% FR for 5 or 10 days until the day of the experiment. The mice that were unable to eat within several hours after feeding and displayed noticeable hypothermia were euthanized from an ethical viewpoint.

### Measurement of blood glucose and ketone body, body temperature, and spontaneous activity

Blood was taken from the tail vein just prior to feeding to determine levels of glucose and ketone body βHB with a OneTouch UltraVue (LifeScan, Tokyo, Japan) and FreeStyle Precision Neo (Abbott, Tokyo, Japan), respectively. Hypoglycemia was defined as the blood glucose level below 50 mg/dL; this level of blood glucose is associated with cognitive impairment, coma, seizure, and/or aberrant behavior in both humans and experimental animals^47^; hypoglycemia-free survival secondary to FR was assessed. Body temperature was recorded just before feeding with a body temperature maintenance system (BWT-100A, Bio Research Center, Nagoya, Japan) connected to a mouse rectal probe (RET-3, Bio Research Center). Daily spontaneous activity was assessed with an infrared ray sensor (NS-AS01, Neuroscience, Tokyo, Japan) at times other than during feeding or taking measurements (09:15–11:45).

### Measurement of body composition and tissue collection

After 5-day FR exposure, fluid status and tissue composition of mice were measured by using an ImpediVET (Bio Research Center) under isoflurane anesthesia followed by sacrifice for blood and tissue collection.

### Alanine-tolerance test

On day 5, mice were injected via the intraperitoneal route with l-alanine (2 g/kg); blood glucose levels were measured before and 30, 60, 90, and 120 min after the injection. In another set of mice, locomotor activity was evaluated between 60 min and 120 min after alanine injection; plasma amino acid levels were determined at 180 min after the injection. Mice were excluded from further analysis for humane reasons if blood glucose levels were <50 mg/dL prior alanine injection.

### Drug treatment

On day 5 of FR, chloroquine (50 mg/kg) was injected intraperitoneally to the young mice and rapamycin (2 mg/kg) to the aged mice. Blood glucose levels were measured before and 60 min after the injections. On day 5, aged FR mice were intraperitoneally injected with Tat-D11 at 20 mg/kg. Blood was collected from the tail vein of the mice before and 60 min after the Tat-D11 injection to measure glucose and βHB levels. The mice were then sacrificed under isoflurane anesthesia for plasma and liver sample collection.

Three sets of aged mice received HET (1.5%)-containing or control pellet chow and were permitted food ad libitum for 4 weeks, after which they were subjected to a 5-day FR (HET was provided during FR at 50 mg/mouse/day in a powdered chow, resulting in approximately equal intake per day both before and during FR). In the first set of mice, the alanine-tolerance test was performed on day 5 of FR to measure blood glucose levels. In the second set of mice, locomotor activity was assessed from 60 min to 120 min after the injection of l-alanine on day 5, and the mice were sacrificed under isoflurane anesthesia for blood collection from the abdominal inferior vena cava at 180 min after the injection. The third set of mice was sacrificed under isoflurane anesthesia on day 5 for tissue collection.

### Transmission electron microscopy

Liver tissues were fixed in 2.5% glutaraldehyde for 24 h at 4 °C, rinsed with 0.1 M phosphate buffer (pH = 7.4), postfixed for 1 h in 1% osmium tetroxide, dehydrated in alcohol, and embedded in epoxy resin. Ultrathin sections stained with uranyl acetate and lead citrate were examined under a Hitachi H-7650H electron microscope (Tokyo, Japan). Scale bars were added to the microscopy images using PowerPoint (Microsoft, Redmond, WA, USA).

### Western blotting

Liver tissues were homogenized with Cell Lysis Buffer (9803, Cell signaling technology, Danvers, MA, USA) containing protease inhibitor cocktail (P8340, Sigma-Aldrich, St. Louis, MO, USA). The homogenates were centrifuged twice at 10,000×*g* at 4 °C for 10 min. Protein aliquots (supernatants of liver tissue homogenates, 10 μg per lane) were separated by sodium dodecyl sulfate–polyacrylamide gel electrophoresis, transferred to a PVDF membrane, and probed with the following primary antibodies: anti-LC3: 1:2500, PM036, MBL International Corporation, Woburn, MA, USA; anti-GAPDH: 1:10,000 and 1:20,000 for western blotting and immunoprecipitation, respectively, 2118, Cell Signaling Technology; anti-Beclin 1: 1:20,000 and 1:50,000 for western blotting and immunoprecipitation, respectively, ab207612 and anti-Bcl-2: 1:1000 and 1:5000 for western blotting and immunoprecipitation, respectively (ab692, Abcam, Cambridge, UK) and secondary antibodies, including anti-rabbit IgG, HRP-linked whole Ab Donkey (1:100,000, NA934-100UL, GE Healthcare, Buckinghamshire, UK), or anti-mouse IgG, HRP-linked F(ab’)2 Fragment Sheep (1:20,000, NA9310V, GE Healthcare). Blots were visualized using the ECL Plus Western Blotting Substrate (32132, Thermo Fisher Scientific, Waltham, MA, USA), scanned by a Typhoon 9410 Imager (GE Healthcare), and quantified with ImageQuant TL (GE Healthcare). All blots or gels derive from the same experiment and they were processed in parallel.

### Immunoprecipitation

Immunoprecipitation was performed according to the manufacturer’s instructions accompanying the Dynabeads protein G immunoprecipitation Kit (10007D, Invitrogen, Waltham, MA, USA); western blotting was performed as described above.

### Real-time quantitative polymerase chain reaction (PCR)

The total RNA was extracted by using an RNeasy Minikit (Qiagen, Valencia, CA, USA) and reverse- transcribed by using the TaqMan High Capacity cDNA Reverse Transcription Kit (Thermo Fisher Scientific). Quantitative PCR assays were performed by using TaqMan Fast Advanced Master Mix (Thermo Fisher Scientific) and TaqMan gene-specific primer/probes (Supplementary Table 3; Thermo Fisher Scientific) on a QuantStudio 7 Flex Real-Time PCR System (Thermo Fisher Scientific). Expression of mRNA was normalized to that of the 18S rRNA gene.

### Glycogen content analysis

The right lobes of the liver were fixed in 10% buffered formalin and embedded in paraffin for histological examination. Sections (5 µm) were stained with hematoxylin and eosin and examined under a BZ-X800 Microscope (Keyence, Osaka, Japan). Scale bars were added to the microscopic images using PowerPoint (Microsoft). To improve visibility, contrast enhancement of the microscopy images was performed using PowerPoint (Microsoft).

The other liver tissues were homogenized on ice with 25 mM citrate buffer, pH 4.2, supplemented with 2.5 g/L NaF, and the supernatants were recovered by centrifugation at 14,000× *g* for 5 min. The glycogen concentration was measured using the EnzyChrom Glycogen Assay Kit (BioAssay Systems, Hayward, CA).

### Measurement of plasma glucose and insulin

The plasma levels of glucose and insulin were measured with the Glucose CII Test Wako Kit (Wako) and the Mouse Insulin ELISA Kit (U-type, Shibayagi, Gunma, Japan).

### Amino acid analysis

Amino acids in plasma and liver samples were derivatized by using an AccQ•Tag Ultra Derivatization Kit (Waters, Milford, MA, USA), and concentrations were determined by an ACQUITY Ultra Performance Liquid Chromatography (UPLC) H-Class system (Waters) equipped with an AccQ•Tag Ultra C18 1.7-μm 2.1 × 100-mm column (Waters) and tunable UV detector (Waters). Amino acid data were processed by using Empower 3 Personal Single System software (Waters). Pathway maps were created by using Visualization and Analysis of Networks software containing Experimental Data V2.6.5 and referenced KEGG pathway databases. The colors in the pathway maps indicated the log2-transformed ratios of each amino acid between groups.

### In vitro autophagy assay

Induction of autophagy was assessed with a HEK293 Autophagy LC3 HiBiT Reporter Cell Line and Detection System (GA1040, Promega Corporation, Madison, WI, USA). Chemical ingredients of HET were purchased or manufactured by Tsumura & Co. (Tokyo, Japan). After the cells (20,000/well) were cultured overnight in 96-well microplates, the medium was removed and treated for 24 h with test compounds that were first dissolved in dimethyl sulfoxide (0.1% at final concentration) and diluted in a medium containing 0.3% fetal bovine serum. HET was suspended by using an agate mortar. Unless otherwise noted, assays were performed according to the manufacturer’s instructions. Values were reported as percentages calculated from the luminescence intensities of vehicle-treated wells (positive controls, 100%) and Bafilomycin A1-treated wells (negative controls, 0%). Compounds that showed inductive activities >120% at either tested concentration were defined as active ingredients of HET.

### Exclusion criteria

Aged mice that developed spontaneous tumors were excluded from the experiment because the tumors introduced confounding variables to this protocol.

### Statistical analyses

Hypoglycemia-free survival was analyzed by the log-rank test. Two-way analysis of variance (ANOVA) followed by the Bonferroni or Tukey post-hoc test was used for time-course analyses. Student’s or the Aspin–Welch *t* test was performed for two-group comparisons, and the Tukey–Kramer, Dunnett, or Steel–Dwass tests were used for multiple-group comparisons. The Mann–Whitney test was used when data distributions were not reliably normally distributed. Chi-square test was used for the analysis of the proportion of mice in locomotor activity assessment. Prism 7 (GraphPad Software, San Diego, CA, USA) and StatLight 2000 (Yukms, Tokyo, Japan) were used to perform data analysis. Plasma amino acids were colored according to a color scale, as described in the text, and *P* values < 0.05 (two-tailed) were considered statistically significant.

### Reporting summary

Further information on research design is available in the Nature Research Reporting Summary linked to this article.

## Supplementary information

Supplementary Information

Reporting Summary

## Data Availability

Data generated in the present study are available from the corresponding author on reasonable request.

## References

[1] Kaiser MJ (2010). Frequency of malnutrition in older adults: a multinational perspective using the mini nutritional assessment. J. Am. Geriatr. Soc..

[2] Yannakoulia M, Ntanasi E, Anastasiou CA, Scarmeas N (2017). Frailty and nutrition: from epidemiological and clinical evidence to potential mechanisms. Metabolism.

[3] Roberts, H. C., Lim, S. E. R., Cox, N. J. & Ibrahim, K. The challenge of managing undernutrition in older people with frailty. *Nutrients* **11**, 10.3390/nu11040808 (2019). 10.3390/nu11040808 PMC6521101 30974825

[4] Herrmann FR, Safran C, Levkoff SE, Minaker KL (1992). Serum albumin level on admission as a predictor of death, length of stay, and readmission. Arch. Intern Med..

[5] Hebuterne X, Schneider S, Peroux JL, Rampal P (1997). Effects of refeeding by cyclic enteral nutrition on body composition: comparative study of elderly and younger patients. Clin. Nutr..

[6] Shizgal HM, Martin MF, Gimmon Z (1992). The effect of age on the caloric requirement of malnourished individuals. Am. J. Clin. Nutr..

[7] Kuma A (2004). The role of autophagy during the early neonatal starvation period. Nature.

[8] Mizushima N, Hara T (2006). Intracellular quality control by autophagy: how does autophagy prevent neurodegeneration?. Autophagy.

[9] Hansen M, Rubinsztein DC, Walker DW (2018). Autophagy as a promoter of longevity: insights from model organisms. Nat. Rev. Mol. Cell Biol..

[10] Heemels MT (2010). Ageing. Nature.

[11] Escobar KA, Cole NH, Mermier CM, VanDusseldorp TA (2019). Autophagy and aging: maintaining the proteome through exercise and caloric restriction. Aging Cell.

[12] Rui L (2014). Energy metabolism in the liver. Compr. Physiol..

[13] Ueno T, Komatsu M (2017). Autophagy in the liver: functions in health and disease. Nat. Rev. Gastroenterol. Hepatol..

[14] Ezaki J (2011). Liver autophagy contributes to the maintenance of blood glucose and amino acid levels. Autophagy.

[15] Takagi A (2016). Mammalian autophagy is essential for hepatic and renal ketogenesis during starvation. Sci. Rep..

[16] Takanashi K (2017). The preventive effect of the traditional Japanese herbal medicine, Hochuekkito, against influenza A virus via autophagy in vitro. Pharmacology.

[17] Yu N, Xiong Y, Wang C (2017). Bu-Zhong-Yi-Qi Decoction, the water extract of Chinese traditional herbal medicine, enhances cisplatin cytotoxicity in A549/DDP cells through induction of apoptosis and autophagy. Biomed. Res. Int..

[18] Takanashi K (2017). Hochuekkito, a Japanese herbal medicine, restores metabolic homeostasis between mitochondrial and glycolytic pathways impaired by influenza A virus infection. Pharmacology.

[19] Shinozuka N, Tatsumi K, Nakamura A, Terada J, Kuriyama T (2007). The traditional herbal medicine Hochuekkito improves systemic inflammation in patients with chronic obstructive pulmonary disease. J. Am. Geriatr. Soc..

[20] Takayama, S. et al. Basic pharmacological mechanisms and clinical evidence of the efficacy of Hochuekkito against infectious diseases and its potential for use against COVID‐19. *Traditional Kampo Med.*10.1002/tkm2.1264 (2020).

[21] Salminen A, Kaarniranta K, Kauppinen A (2012). Inflammaging: disturbed interplay between autophagy and inflammasomes. Aging.

[22] Gelegen C, Collier DA, Campbell IC, Oppelaar H, Kas MJ (2006). Behavioral, physiological, and molecular differences in response to dietary restriction in three inbred mouse strains. Am. J. Physiol. Endocrinol. Metab..

[23] Izumida Y (2013). Glycogen shortage during fasting triggers liver-brain-adipose neurocircuitry to facilitate fat utilization. Nat. Commun..

[24] Parrilla R (1978). Flux of metabolic fuels during starvation in the rat. Pflugers Arch..

[25] Rognstad R (1979). Rate-limiting steps in metabolic pathways. J. Biol. Chem..

[26] Yoon JC (2001). Control of hepatic gluconeogenesis through the transcriptional coactivator PGC-1. Nature.

[27] Iwai-Kanai E (2008). A method to measure cardiac autophagic flux in vivo. Autophagy.

[28] Gomes LC, Di Benedetto G, Scorrano L (2011). During autophagy mitochondria elongate, are spared from degradation and sustain cell viability. Nat. Cell Biol..

[29] Jung CH, Ro SH, Cao J, Otto NM, Kim DH (2010). mTOR regulation of autophagy. FEBS Lett..

[30] Kihara A, Kabeya Y, Ohsumi Y, Yoshimori T (2001). Beclin-phosphatidylinositol 3-kinase complex functions at the trans-Golgi network. EMBO Rep..

[31] Shoji-Kawata S (2013). Identification of a candidate therapeutic autophagy-inducing peptide. Nature.

[32] Pattingre S (2005). Bcl-2 antiapoptotic proteins inhibit Beclin 1-dependent autophagy. Cell.

[33] Matsunaga K (2009). Two Beclin 1-binding proteins, Atg14L and Rubicon, reciprocally regulate autophagy at different stages. Nat. Cell Biol..

[34] Fernandez AF (2018). Disruption of the Beclin 1-BCL2 autophagy regulatory complex promotes longevity in mice. Nature.

[35] Molpeceres V (2007). Melatonin is able to reduce the apoptotic liver changes induced by aging via inhibition of the intrinsic pathway of apoptosis. J. Gerontol. A Biol. Sci. Med Sci..

[36] Mach J (2015). The effect of aging on mitochondrial and cytosolic hepatic intrinsic death pathway and apoptosis associated proteins in Fischer 344 rats. Exp. Gerontol..

[37] Yakubo S, Ozawa Y, Kanmatsuse K (2000). Clinical experience of Hochu-Ekki-to for symptoms indicating a state of Ki-deficiency and low body temperature. Jpn. J. Orient. Med..

[38] Satoh N (2005). A randomized double blind placebo-controlled clinical trial of Hochuekkito, a traditional herbal medicine, in the treatment of elderly patients with weakness N of one and responder restricted design. Phytomedicine.

[39] Tatsumi K (2009). Hochuekkito improves systemic inflammation and nutritional status in elderly patients with chronic obstructive pulmonary disease. J. Am. Geriatr. Soc..

[40] Wang S (2016). 6-Gingerol induces autophagy to protect HUVECs survival from apoptosis. Chem. Biol. Interact..

[41] Huang Z, Liu Y, Huang X (2018). Formononetin may protect aged hearts from ischemia/reperfusion damage by enhancing autophagic degradation. Mol. Med. Rep..

[42] Wang Y, Ren Q, Zhang X, Lu H, Chen J (2018). Neuroprotective mechanisms of calycosin against focal cerebral ischemia and reperfusion injury in rats. Cell Physiol. Biochem..

[43] Dong X (2017). Maslinic acid promotes autophagy by disrupting the interaction between Bcl2 and Beclin1 in rat pheochromocytoma PC12 cells. Oncotarget.

[44] Liu J (2014). Oleanolic acid induces protective autophagy in cancer cells through the JNK and mTOR pathways. Oncol. Rep..

[45] Guo J (2014). Ginsenoside compound K promotes beta-amyloid peptide clearance in primary astrocytes via autophagy enhancement. Exp. Ther. Med.

[46] Owen OE, Felig P, Morgan AP, Wahren J, Cahill GF (1969). Liver and kidney metabolism during prolonged starvation. J. Clin. Investig..

[47] Cryer PE (2007). Hypoglycemia, functional brain failure, and brain death. J. Clin. Investig..

