# Supplementary material for: Bcl-2-dependent autophagy disruption during aging impairs amino acid utilization that is restored by hochuekkito
Source: NPJ Aging Mech Dis. 2021 Jul 1;7:13. doi: 10.1038/s41514-021-00065-8 (PMC8249599; doi:10.1038/s41514-021-00065-8)
Supplement: Supplementary file 1 — Supplementary Information [file 41514_2021_65_MOESM1_ESM.pdf]

Supplementary Table 1. Baseline characteristics of young and aged mice

	**Young**	**Aged**
Body weight (g)	25.2 ± 0.4 (n = 8)	33.0 ± 0.3 (n = 13) ***
Spontaneous activity (counts/day)	6203 ± 502 (n = 10)	2767 ± 271 (n = 13) ***
Body temperature (ºC)	35.7 ± 0.3 (n = 8)	35.6 ± 0.2 (n = 13)
Blood glucose (mg/dL)	141.0 ± 8.0 (n = 8)	127.4 ± 3.2 (n = 13)
Blood β-hydroxybutyrate (mM)	0.53 ± 0.05 (n = 8)	0.27 ± 0.02 (n = 13) ***
Plasma insulin	322.9 ± 31.6 (n = 12)	1598.7 ± 405.8 (n = 10) *

Data are presented as the mean ± SEM. *$p < 0.05$, ***$p < 0.001$ *vs*. young mice by Student's or Aspin–Welch's *t*-test.

Supplementary Table 2. Baseline characteristics of HET-treated aged mice

	**Aged control**	**Aged control + HET**
Body weight (g)	36.5 ± 0.9 (n = 11)	35.5 ± 0.6 (n = 13)
Epididymal fat weight (g)	1.15 ± 0.13 (n = 11)	0.94 ± 0.11 (n = 13)
Gastrocnemius muscle weight (mg)	147.8 ± 6.5 (n = 11)	152.2 ± 2.4 (n = 13)
Soleus muscle weight (mg) (both legs combined)	17.7 ± 3.1 (n = 11)	13.6 ± 0.7 (n = 13)
Spontaneous activity (counts/day)	4524 ± 586 (n = 16)	3862 ± 387 (n = 14)
Body temperature (ºC)	37.1 ± 0.2 (n = 16)	36.9 ± 0.3 (n = 11)
Plasma glucose (mg/dL)	213.1 ± 10.1 (n = 11)	208.4 ± 7.1 (n = 13)
Plasma insulin (pg/mL)	1989.1 ± 335.2 (n = 11)	1451.6 ± 181.0 (n = 13)

Data are presented as the mean ± SEM.

Supplementary Table 3. Primers used for real-time PCR

Target	Gene name	Assay ID
18S	Eukaryotic 18S rRNA	Hs99999901_s1
G6Pase	glucose-6-phosphatase, catalytic	Mm00839363_m1
PEPCK	phosphoenolpyruvate carboxykinase 1, cytosolic	Mm01247058_m1
PGC-1α	peroxisome proliferative activated receptor, gamma, coactivator 1 alpha	Mm01208835_m1
MuRF1	tripartite motif-containing 63	Mm01185221_m1
Atrogin-1	F-box protein 32	Mm00499523_m1
LC3	microtubule-associated protein 1 light chain 3 beta	Mm00782868_sH
P62	sequestosome 1	Mm00448091_m1
Atg5	autophagy related 5	Mm01187303_m1
Atg7	autophagy related 7	Mm00512209_m1
Atg12	autophagy related 12	Mm00503201_m1
Bnip3	BCL2/adenovirus E1B interacting protein 3	Mm01275600_g1
Pink1	PTEN induced putative kinase 1	Mm00550827_m1
Becn1	beclin1	Mm01265461_m1
Bcl2	B cell leukemia/lymphoma 2	Mm00477631_m1
Rubicon	Rubicon	Mm00553869_m1
Pparα	peroxisome proliferator activated receptor α	Mm00440939_m1
Hmgcs2	3-hydroxy-3-methylglutaryl coenzyme A synthase 2	Mm00550050_m1
Slc1a4	solute carrier family 1 (glutamate/neutral amino acid transporter), member 4	Mm01223875_m1
Slc1a5	solute carrier family 1 (neutral amino acid transporter), member 5	Mm00436603_m1
Mpc1	mitochondrial pyruvate carrier 1	Mm01316203_g1
Mpc2	mitochondrial pyruvate carrier 2	Mm00770995_m1
Mfn1	mitofusin 1	Mm00612599_m1
Mfn2	mitofusin 2	Mm00500120_m1
Fis1	fission 1 (mitochondrial outer membrane) homolog (yeast)	Mm00481580_m1
Opa1	optic atrophy 1	Mm01349707_g1
Cxcl5	chemokine (C-X-C motif) ligand 5	Mm00436451_g1
Il1b	interleukin 1 beta	Mm00434228_m1
Il6	interleukin 6	Mm00446190_m1
Tnf	tumor necrosis factor	Mm00443258_m1
Ccl2	chemokine (C-C motif) ligand 2/monocyte chemotactic protein 1 (MCP-1)	Mm00441242_m1
Ptgs2	prostaglandin-endoperoxide synthase 2/ cyclooxygenase-2 (Cox2)	Mm00478374_m1
Nos2	nitric oxide synthase 2, inducible	Mm00440502_m1

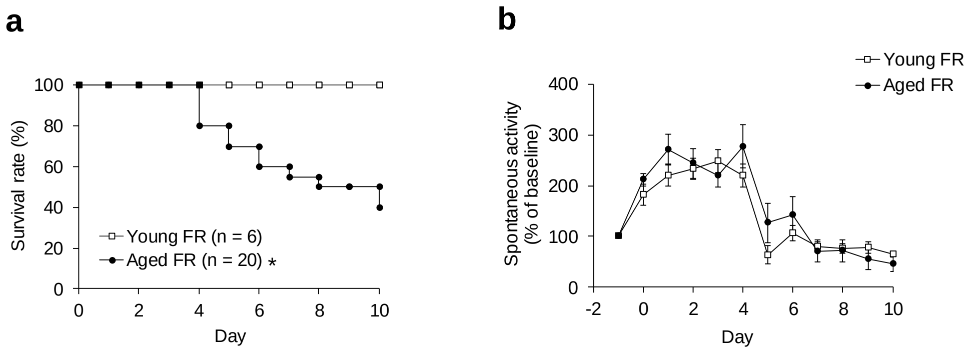

Supplementary Fig. 1. Survival rate and spontaneous activity in food-restricted (FR) young and aged mice.
a Overall survival rate. * $p < 0.05$ *vs*. young FR mice by log-rank test. **b** Percent change in daily spontaneous activity from baseline (day −1). Data are presented as mean ± SEM. Young FR (n = 10), aged FR (n= 7– 13).

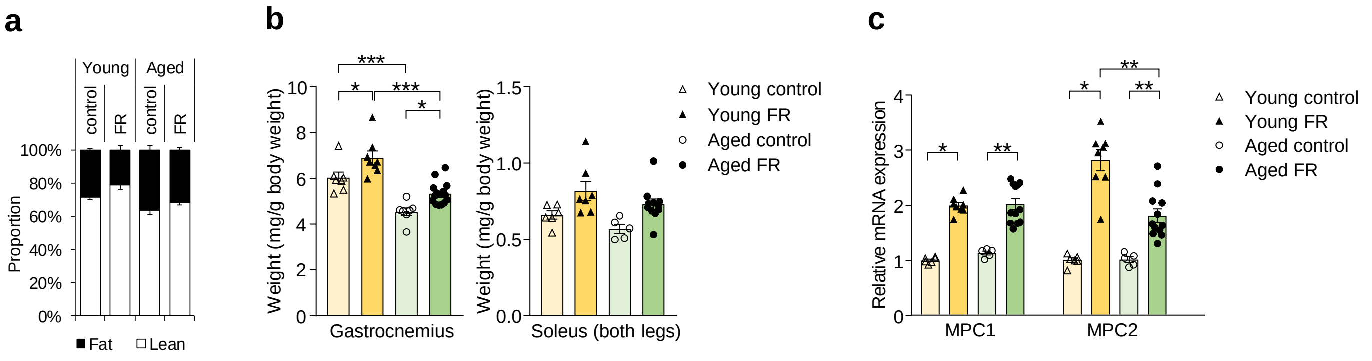

Supplementary Fig. 2. Body composition and hepatic gene expression in 5-day-food-restricted (FR) young and aged mice.

a Body composition. Young control (n = 6), young FR (n = 6), aged control (n = 5), aged FR (n = 14). **b** Muscle weights per body weight. Gastrocnemius; young control (n = 7), young FR (n = 8), aged control (n = 7), aged FR (n = 15). Soleus; young control (n = 6), young. FR (n = 8), aged control (n = 5), aged FR (n = 12). **c** Hepatic gene expression of mitochondrial pyruvate carrier (MPC) 1 and MPC2. Young control (n = 6), young FR (n = 8), aged control (n = 5), aged FR (n = 12). **p* < 0.05, ***p* < 0.01, ****p* < 0.001 by the Tukey–Kramer or Steel–Dwass test. Data are presented as mean ± SEM.

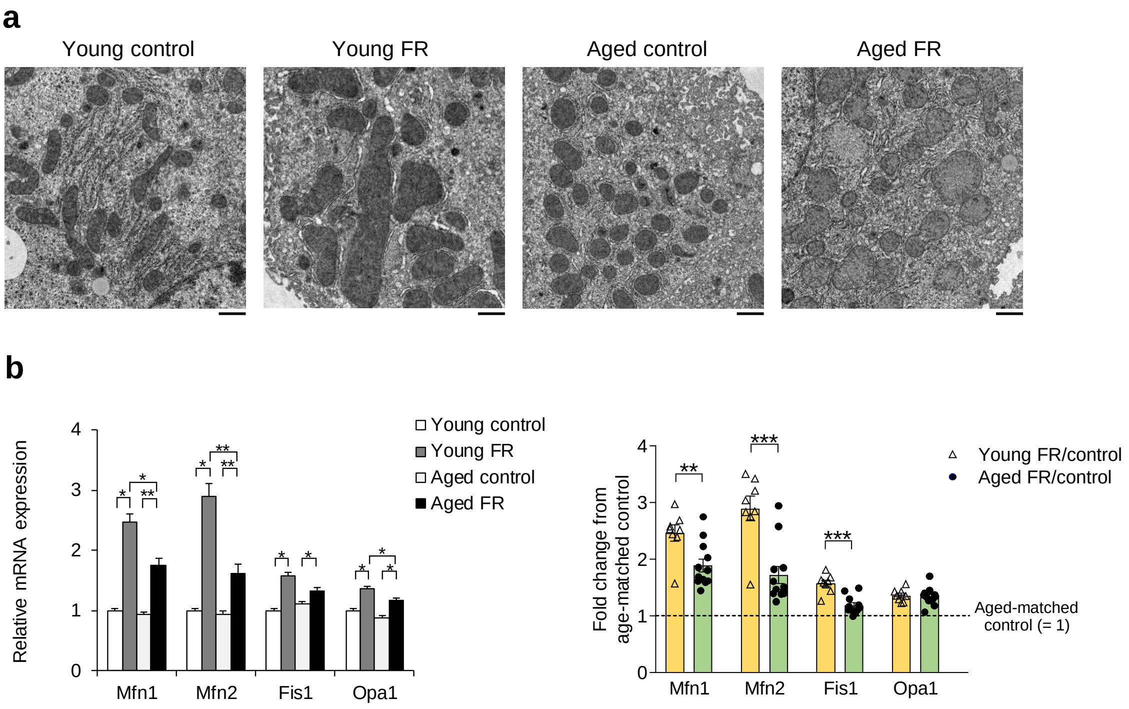

Supplementary Fig. 3. Mitochondrial morphologic change in 5-day-food-restricted (FR) young and aged mice.

a Electron microscope images of hepatic mitochondria. Bar = 1 μm. **b** Hepatic gene expression of mitochondrial fusion- and fission-related peptides. *$p < 0.05$, **$p < 0.01$, ***$p < 0.001$ by the Steel–Dwass test (left panel) or Student's *t*-test (right panel); young control (n = 6), young FR (n = 8), aged control (n = 5), aged FR (n = 12). Data are presented as mean ± SEM.

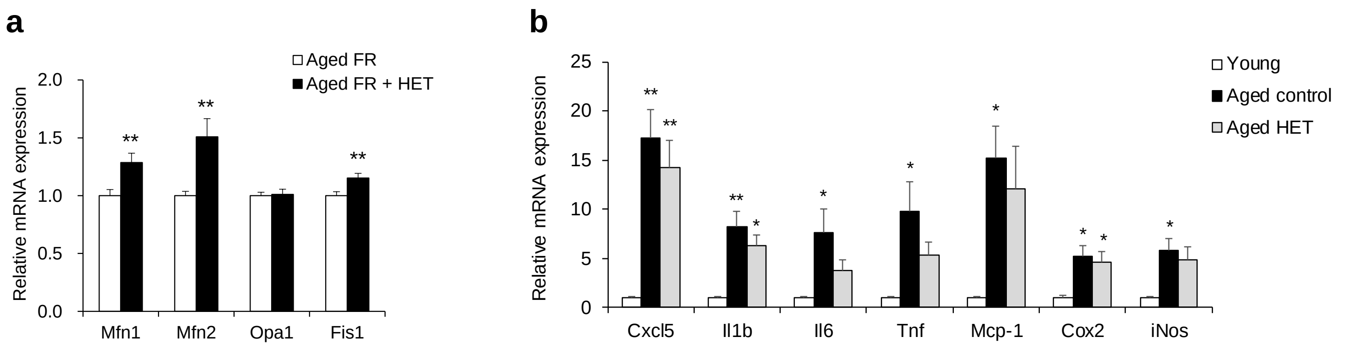

Supplementary Fig. 4. Effect of treatment of hochuekkito (HET) on hepatic gene expression in aged mice.

a Expression of gene encoding mitochondrial fusion- and fission-related peptides in the liver in aged mice on 5-day food restriction (FR). Mice were fed HET (1.5%)-containing pellet chow *ad libitum* for 4 weeks and then subjected to 5-day FR. **$p < 0.01$ *vs*. aged FR mice by Aspin–Welch's *t*-test; control (n = 14), HET (n = 13). **b** Expression of gene encoding inflammatory-related peptides in the liver in aged mice after 4-week HET-containing pellet chow feeding. *$p < 0.05$, ** $p < 0.01$ *vs*. young mice by the Tukey's test; young (n = 8), aged control (n = 11), aged HET (n = 13). Data are presented as mean ± SEM.

Fig. 5c

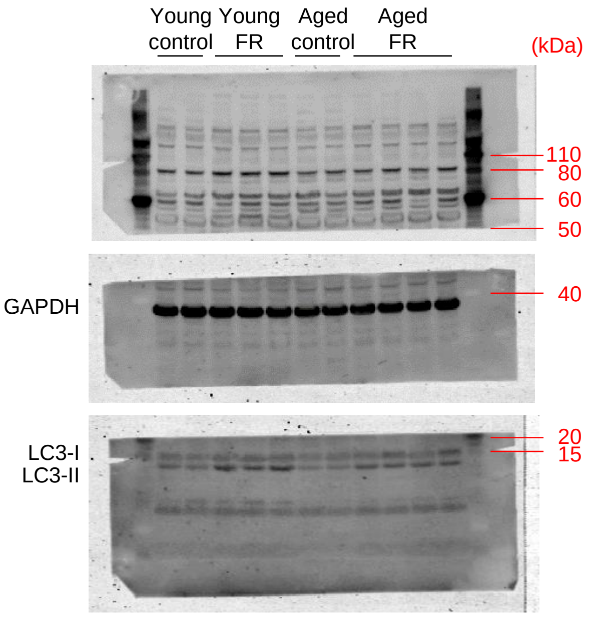

Fig. 7b

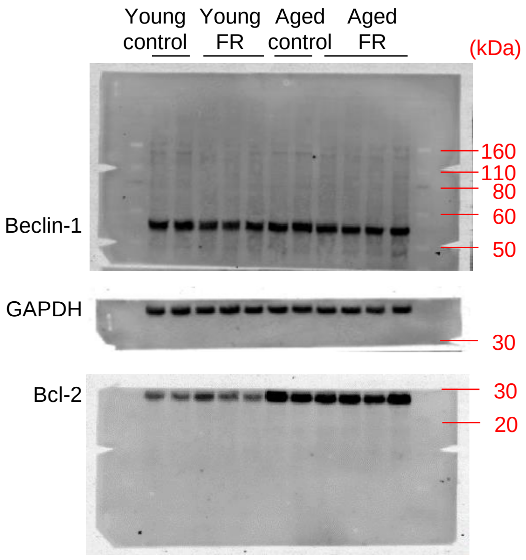

Fig. 7c

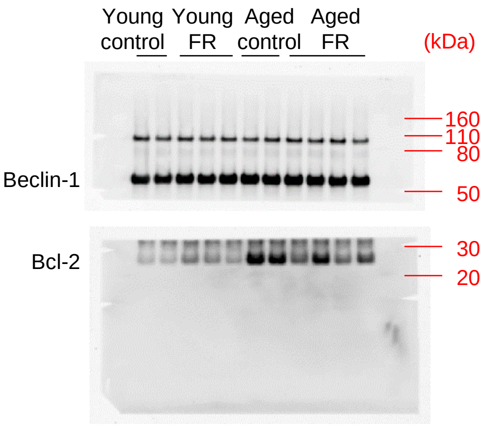

Supplementary Fig. 5. Full-length images of blots.
