# Supplementary material for: Bcl-2-dependent autophagy disruption during aging impairs amino acid utilization that is restored by hochuekkito
Source: NPJ Aging Mech Dis. 2021 Jul 1;7:13. doi: 10.1038/s41514-021-00065-8 (PMC8249599; doi:10.1038/s41514-021-00065-8)
Supplement: Supplementary file 2 — Reporting Summary [file 41514_2021_65_MOESM2_ESM.pdf]

nature research

Corresponding author(s): Hiroshi Takeda

Last updated by author(s): Mar 16, 2021

Reporting Summary

Nature Research wishes to improve the reproducibility of the work that we publish. This form provides structure for consistency and transparency in reporting. For further information on Nature Research policies, see our Editorial Policies and the Editorial Policy Checklist.

Statistics

For all statistical analyses, confirm that the following items are present in the figure legend, table legend, main text, or Methods section.

n/a	Confirmed	
☐	☒	The exact sample size (n) for each experimental group/condition, given as a discrete number and unit of measurement
☐	☒	A statement on whether measurements were taken from distinct samples or whether the same sample was measured repeatedly
☐	☒	The statistical test(s) used AND whether they are one- or two-sided<br>*Only common tests should be described solely by name; describe more complex techniques in the Methods section.*
☒	☐	A description of all covariates tested
☐	☒	A description of any assumptions or corrections, such as tests of normality and adjustment for multiple comparisons
☐	☒	A full description of the statistical parameters including central tendency (e.g. means) or other basic estimates (e.g. regression coefficient) AND variation (e.g. standard deviation) or associated estimates of uncertainty (e.g. confidence intervals)
☐	☒	For null hypothesis testing, the test statistic (e.g. F, t, r) with confidence intervals, effect sizes, degrees of freedom and P value noted<br>*Give P values as exact values whenever suitable.*
☒	☐	For Bayesian analysis, information on the choice of priors and Markov chain Monte Carlo settings
☒	☐	For hierarchical and complex designs, identification of the appropriate level for tests and full reporting of outcomes
☒	☐	Estimates of effect sizes (e.g. Cohen's d, Pearson's r), indicating how they were calculated

Our web collection on statistics for biologists contains articles on many of the points above.

Software and code

Policy information about availability of computer code

Data collection	No software was used.
Data analysis	No software was used.

For manuscripts utilizing custom algorithms or software that are central to the research but not yet described in published literature, software must be made available to editors and reviewers. We strongly encourage code deposition in a community repository (e.g. GitHub). See the Nature Research guidelines for submitting code & software for further information.

Data

Policy information about availability of data

All manuscripts must include a data availability statement. This statement should provide the following information, where applicable:

- Accession codes, unique identifiers, or web links for publicly available datasets
- A list of figures that have associated raw data
- A description of any restrictions on data availability

Data generated in the present study are available from the corresponding author on reasonable request.

Field-specific reporting

Please select the one below that is the best fit for your research. If you are not sure, read the appropriate sections before making your selection.

☒ Life sciences ☐ Behavioural & social sciences ☐ Ecological, evolutionary & environmental sciences

For a reference copy of the document with all sections, see nature.com/documents/nr-reporting-summary-flat.pdf

Life sciences study design

All studies must disclose on these points even when the disclosure is negative.

Sample size	Sample size was determined based on analysis of the data obtained in preliminary and previous experiments in our laboratory, taking into consideration data variation and minimizing the number of animals used.
Data exclusions	Aged mice that developed spontaneous tumors were excluded from the experiment because the tumors introduced confounding variables into the study.
Replication	Most of the experiments were conducted in duplicates or after smaller-scale evaluation. Similar results were obtained in the first and second set of experiments. In time-course experiments, we also confirmed similar results at the main time point.
Randomization	Mice fed ad libitum were randomly allocated into groups to minimize body weight variation among different groups .<br>Mice subjected to food restriction were randomly allocated into groups to minimize blood glucose and body temperature variation among different groups.
Blinding	Since majority of the assessments in our study were performed using apparatuses and not rating or scoring methods, we did not use blinding approach. Histological examinations were conducted without blinding by a specialist in pathology.

Reporting for specific materials, systems and methods

We require information from authors about some types of materials, experimental systems and methods used in many studies. Here, indicate whether each material, system or method listed is relevant to your study. If you are not sure if a list item applies to your research, read the appropriate section before selecting a response.

Materials & experimental systems

n/a	Involved in the study	
☐	☒	Antibodies
☒	☐	Eukaryotic cell lines
☒	☐	Palaeontology and archaeology
☐	☒	Animals and other organisms
☒	☐	Human research participants
☒	☐	Clinical data
☒	☐	Dual use research of concern

Methods

n/a	Involved in the study	
☒	☐	ChIP-seq
☒	☐	Flow cytometry
☒	☐	MRI-based neuroimaging

Antibodies

Antibodies used	anti-LC3 (MBL International Corporation, Cat# PM036, Lot# 032)<br>anti-GAPDH (Cell Signaling Technology, Cat# 2118, Lot# 8)<br>anti-Beclin 1 (Abcam, Cat# ab207612, Lot# GR3198372-5 for WB, Lot# GR3254931-3 for IP)<br>anti-Bcl-2 (Abcam, Cat# ab692, Lot# GR3266609-2)<br>anti-rabbit IgG, HRP-linked whole Ab Donkey (GE Healthcare, Cat# NA934, Lot# 9653375)<br>anti-Mouse IgG, HRP-linked F(ab’)2 Fragment Sheep (GE Healthcare, Cat# NA9310V, Lot# 9578359)
Validation	anti-LC3; https://www.mblintl.com/products/pm036/<br>anti-GAPDH; https://www.cellsignal.com/products/primary-antibodies/gapdh-14c10-rabbit-mab/2118<br>anti-Beclin 1; https://www.abcam.com/beclin-1-antibody-epr19662-ab207612.html<br>anti-Bcl-2; https://www.abcam.com/bcl-2-antibody-100d5-ab692.html

Animals and other organisms

Policy information about studies involving animals; ARRIVE guidelines recommended for reporting animal research

Laboratory animals	Male C57BL/6J mice aged 9 weeks (young group) and 23–26 months (aged group) were used.

Wild animals	This study did not involve wild animals.
Field-collected samples	This study did not involve field collected samples.
Ethics oversight	All animal experiments were conducted in compliance with the ARRIVE guidelines. This study was approved by and conducted according to the guidelines of the experimental animal ethics committee of Tsumura & Co. (Tokyo, Japan; permit no. 15-037, 15-074, 16-069, 17-003, and 17-036). Their animal experimentation facility is accredited by the Japan Health Sciences Foundation (http://www.jhsf.or.jp/).

Note that full information on the approval of the study protocol must also be provided in the manuscript.
